# New-onset childhood extensive cutaneous lichen planus following asymptomatic COVID-19 infection: report of a case

**DOI:** 10.1590/1984-0462/2024/42/2023004

**Published:** 2023-10-23

**Authors:** Fatemeh Ansari Asl, Mozhdeh Sepaskhah, Marzie Rassafian, Fatemeh Sari Aslani, Farideh Jowkar

**Affiliations:** a Department of Dermatology, School of Medicine, Shiraz University of Medical Sciences, Shiraz, Iran.; b Molecular Dermatology Research Center, Department of Dermatology, School of Medicine, Shiraz University of Medical Sciences, Shiraz, Iran.; c Molecular Dermatology Research Center, Department of Pathology, School of Medicine, Shiraz University of Medical Sciences, Shiraz, Iran.

**Keywords:** Lichen planus, COVID-19, Child, preschool, Skin diseases, Therapy, Líquen plano, COVID-19, Criança pré-escolar, Dermatopatias, Terapia

## Abstract

**Objective:**

The objective of this study was to describe a case of cutaneous lichen planus (LP) that appeared following COVID-19 infection.

**Case description:**

We report a case of extensive cutaneous classic familial LP in a 4-year-old male child after an asymptomatic serologically confirmed COVID-19 infection. The patient developed intensely itchy, purple, flat-topped papules and plaques, mainly on the dorsal surface of the hands, feet, forearms, and shins. Histopathological examination of the skin biopsy showed vacuolar and apoptotic degeneration of the basal cell layer with a band-like lymphocyte infiltrate at the dermo-epidermal junction and confirmed the diagnosis of LP.

**Comments:**

LP could be considered among the differential diagnoses of pediatric post-COVID inflammatory skin lesions, either in the patients recovering from COVID-19 infection or in the suspicious asymptomatic cases in close contact with COVID-19-infected patients.

## RESUMO

**Objetivo:**

Descrever um caso de líquen plano cutâneo (LP) após infecção por COVID-19.

**Descrição do caso:**

Relatamos um caso de LP familiar clássico extenso cutâneo em uma criança de quatro anos de idade após uma infecção por COVID-19 assintomática e sorologicamente confirmada. O paciente desenvolveu pápulas e placas intensamente pruriginosas, roxas e achatadas, principalmente na superfície dorsal das mãos, pés, antebraços e canelas. O exame histopatológico da biópsia de pele mostrou degeneração vacuolar e apoptótica da camada basal com infiltrado de linfócitos em faixa na junção dermoepidérmica e confirmou o diagnóstico de líquen plano.

**Comentários:**

O líquen plano pode ser considerado entre os diagnósticos diferenciais de lesões cutâneas inflamatórias pós-COVID pediátricas, tanto em pacientes em recuperação de infecção por COVID-19 quanto em casos assintomáticos suspeitos em contato próximo com pacientes infectados por COVID-19.

## INTRODUCTION

Skin manifestations are among the frequently described signs of COVID-19 infection in adults and children.^1–5^ These cutaneous features include chilblain-like lesions, acute urticaria, erythema multiforme-like eruption, papulovesicular eruption, and non-specific skin lesions.^2^ Besides the skin lesions that develop during active disease, COVID-19 infection can rise or exacerbate some skin diseases.^6–8^ There are few reports of skin lesions during or after asymptomatic COVID-19 infection.^9^

Lichen planus (LP) is a rare complication of COVID-19 infection^10^ and is mainly reported after COVID-19 vaccination.^11^ Some post-COVID LP lesions involved only oral mucosa,^12,13^ and the other cases were cutaneous and/or mucocutaneous LP.^10,14,15^

LP may occur in children, although it is not a common skin disorder in this age group.^16^ Herein, we report the appearance of extensive cutaneous LP following an asymptomatic COVID-19 infection in a child.

## CASE REPORT

**Figure 1:**
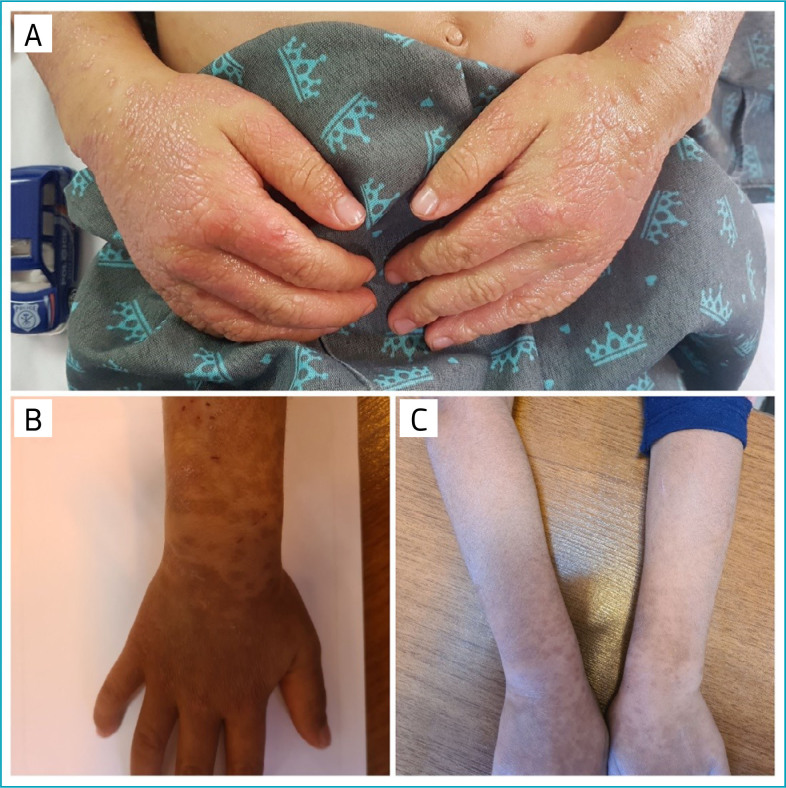
Clinical features. (A) Pruritic violaceous erythematous flat-topped papules and plaques on the upper extremities after asymptomatic COVID-19 infection. (B) Evolution of the lesions 3 months after treatment. (C) Post-inflammatory hyperpigmentation in 10 months follow-up.

A 4-year-old male child was admitted to the dermatology ward of our referral hospital in Shiraz, South of Iran, due to generalized, intensely itchy lesions that started about 3 months before admission. The lesions were violaceous, flat-topped papules and plaques on the trunk, extremities, and face. The skin lesions gathered especially on the dorsal surface of hands, feet, forearms, and shins (Figure 1A). There was no sign of mucosal, hair, or nail involvement.

The patient was a known case of single kidney disease and hypoglycemia since birth without recent significant problems. His mother had a history of localized cutaneous LP controlled by topical corticosteroid. He had not started any new medications recently and was not taking any drugs chronically. He had close household contact with a confirmed case of COVID-19 infection (his mother) about 2 weeks before the development of the lesions, without any symptoms of upper or lower respiratory tract infection, gastrointestinal infection, skin rash, or fever.

**Figure 2:**
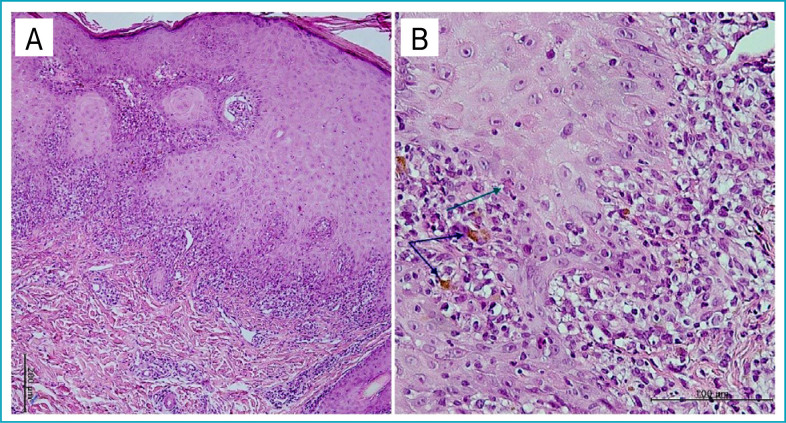
Histopathologic features. (a) Hyperorthokeratosis, focal hypergranulosis, irregular acanthosis, vacuolar degeneration of the basal cell layer, and a band-like lymphocyte infiltrate at the dermo-epidermal junction (H&E stain, 100X). (b) Colloid bodies (green arrow) in the basal layer and pigment incontinence (blue arrows) in the papillary dermis (H&E stain, 400X).

The clinical differential diagnoses included LP and psoriasis. Histopathological examination of the skin punch biopsy revealed hyperkeratosis without parakeratosis, focal increase in the granular cell layer, irregular acanthosis, and vacuolar degeneration of the basal cell layer with a band-like lymphocyte infiltrate at the dermo-epidermal junction. Colloid bodies were present at the lower level of the epidermis (Figure 2). Hence, the diagnosis of LP was confirmed.

Laboratory work-up revealed normal results of complete blood count, liver function tests, blood urea nitrogen, creatinine, fasting blood sugar, and C-reactive protein. Serological examination showed high titers of IgM for COVID-19.

The patient was treated with systemic corticosteroid (prednisolone 7.5 mg daily, increased to 12.5 mg daily), clobetasol propionate ointment at night, and topical tacrolimus 0.03% ointment twice daily (for face and flexural areas). Oral sedative antihistamines were prescribed to improve pruritus and disturbed sleep. After 1 month of follow-up, the skin lesion improved significantly (Figure 1B). Hence, we tapered prednisolone for 3 months and then discontinued it (Figure 1B). No relapse occurred in 10 months of follow-up (Figure 1C).

The patient's parent signed written informed consent and permitted the publication of the case and photography without identifying data. Maintaining the patient's confidentiality is guaranteed by researchers. The institutional ethics committee approved the case report (ethics code: IR.SUMS.MED.REC.1401.403).

## DISCUSSION

COVID-19 is a multisystem disease, and several skin manifestations are described during and after this infection.^1^ Several cases of newly developed or exacerbated skin disorders are reported after COVID-19 infection, including psoriasis, pityriasis rosea, vasculitis, alopecia areata, vitiligo, bullous pemphigoid, and many other skin diseases.^6–8^ Rarely, inflammatory skin lesions are reported after asymptomatic, serologically-confirmed COVID-19 infection.^9^

LP is a T-cell-mediated autoimmune dermatologic disorder. The development of autoimmunity mostly depends on a combination of factors such as genetic predisposition, inadequate immune response, and environmental triggers such as viral infections.^17^ While the causal effect of microbial agents in the pathogenesis of LP is debated,^18^ the association of LP with several viruses (e.g., hepatitis C virus, human herpes virus-7, and Ebstein-Barr virus) is disclosed.^19–21^

New-onset LP has been sporadically reported after COVID-19 infection.^10,12,14,15^ More LP cases are reported after COVID-19 vaccination compared to COVID-19 infection.^11,13^ The post-COVID LP lesions may involve skin,^11^ oral mucosae,^9,10^ or both.^10,15^ To the best of our knowledge, all reported post-COVID LP cases were adults.^10,12-15^ One of these cases presented with annular LP.^10^ Sood et al.^22^ proposed the hypothesis of the pathogenesis of post-COVID oral LP. They discussed the related target receptors, abnormal T-cell responses, elevated levels of cytokines, disrupted immune permeability barrier, and vitamin D deficiency as the possible causes of post-COVID oral LP.^22^

LP is not a common dermatologic disorder in the pediatric age group. Most pediatric LP cases occur in school-aged children.^16^ The clinical presentation of classic LP is similar in adults and children and includes pruritic, polygonal, flat-topped, purple papules and plaques with fine scale and Wickham's striae. The lesions may involve any body area but have a predilection for flexural parts of extremities, dorsa of the hands, shins, trunk, and sacral area.^16^ Oral involvement is less common in pediatric LP compared to adult LP.^23^

Pediatric LP is usually treated with topical corticosteroids in the limited disease and systemic corticosteroids in the more extensive disease. Phototherapy or systemic immunomodulators (e.g., dapsone, antimalarial drugs, methotrexate, and cyclosporine) are other therapeutic choices in the extensive pediatric LP.^23^

Our case was a child of pre-school age that developed extensive, severe classic cutaneous familial LP following asymptomatic serologically confirmed COVID-19 infection. He was treated successfully with topical and systemic corticosteroids. This case is unusual in LP's age, severity, and presentation setting.

To the best of our knowledge, this is the first case of post-COVID LP in the pediatric age group and the first case of LP following asymptomatic COVID-19 infection. Thus, it is essential to consider LP among the differential diagnoses of pediatric post-COVID inflammatory skin lesions, either in the patients recovering from COVID-19 infection or in the suspicious asymptomatic cases that have been in close contact with COVID-19-infected patients.
